# Supplementary material for: Production of High-Quality Wheat Sprouts of Strong Antioxidant Capacity: Process Optimization and Regulation Mechanism of Red Light Treatment
Source: Foods. 2024 Aug 27;13(17):2703. doi: 10.3390/foods13172703 (PMC11395093; doi:10.3390/foods13172703)
Supplement: Supplementary file 1 [file foods-13-02703-s001.zip › foods-3165953-supplementary.pdf]

SUPPLEMENT FIGURE CAPTIONS

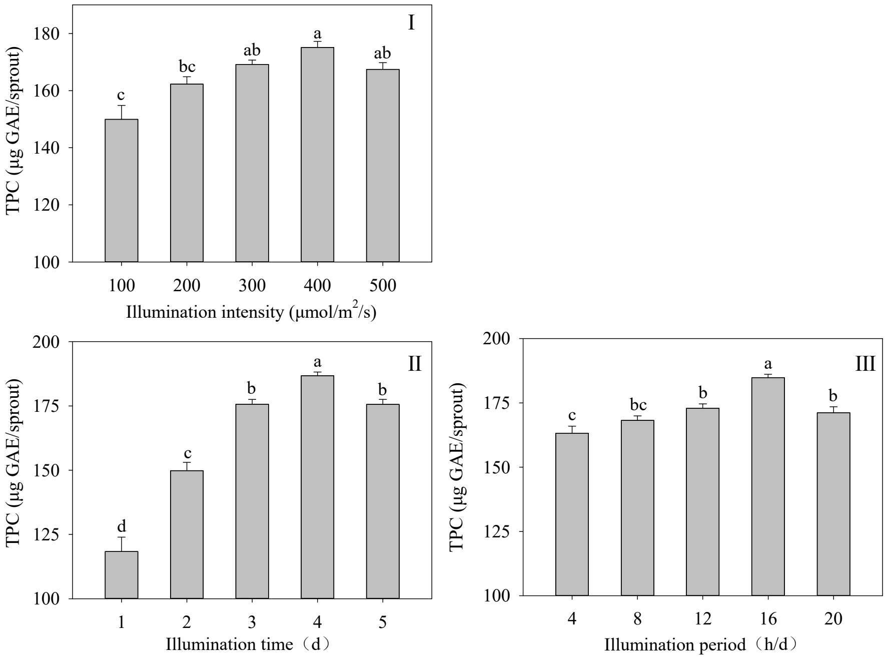

Figure S1. Effect of illumination intensity (I), illumination period (II), and illumination period (III) on total phenolic content in wheat seedlings. Each data point represents the average (average ± SD) of three independent biological replicates. Lowercase letters reflect significant differences (p < 0.05) in indicators between treatments at a given germination time using Tukey's test. TPC represents the total phenolic content, and it was reported to be gallic acid equivalents.

Table S1. Encoded and real levels for the germination process of wheat sprout and the total phenolic content of each treatment group

Independent variables	Levels		
	-1	0	1
X_1: Illumination intensity ($\mu mol/m^2/s$)	300	400	500
X_2: Illumination time (d)	3	4	5
X_3: Illumination period (h/d)	12	16	20

No.	X_1: Illumination intensity ($\mu mol/m^2/s$)	X_2: Illumination time (d)	X_3: Illumination period (h/d)	Y: TPC[1] (μg GAE/sprout)
1	-1	-1	0	166.78±1.81
2	1	-1	0	169.69±2.12
3	-1	1	0	170.62±1.36
4	1	1	0	171.20±2.14
5	-1	0	-1	177.45±1.67
6	1	0	-1	178.50±4.54
7	-1	0	1	179.73±1.64
8	1	0	1	182.33±0.76
9	0	-1	-1	169.23±1.43
10	0	1	-1	173.78±1.30
11	0	-1	1	172.77±1.20
12	0	1	1	173.49±1.17
13	0	0	0	184.79±1.21
14	0	0	0	185.07±0.89
15	0	0	0	185.02±1.13
16	0	0	0	185.14±0.97
17	0	0	0	184.58±1.55

[1] TPC represents the total phenolic content, and it was reported to be gallic acid equivalents.

Table S2. The sequences of the primers utilized in the investigation

Gene Name	Forward Primer (5′ - 3′)	Reverse Primer (5' - 3')
Actin	AGCGGTCGAACAACTGGTA	AAACGAAGGATAGCATGAGGAAGC
TaPOD	GACCAGGTGCTCTTCAACAACGAC	TAGCCGTAGGTCAATCACGAGTTC
TaSOD	GAGTAGAAATCCGCTCCCGAC	GAGGCAGGAACCTGGAAGAGA
TaAPX	CAGCAATGTGTGAGGCTTGGC	CAATGATGACACCGTCGGTGG
TaCAT	TGCCTGTGTTTTTTATCCGA	ACCGTCCATGTGCCTGTAGT
TaPAL	CACCACCCTGGACAGATTG	TGAGGCGAAGTGCGGAG
Ta4CL	ACATTACACAAGCAGGAAGAACC	CACTCAGCCAGCCAGCAG
TaC3H	ATTGACGAAGAAGGGCAG	GGACACAGCCATCTCAAGT
TaC4H	CAGCCTCCACATCCTCAAG	CTTAGGACGAGCGAACAATC
TaF5H	AGCTCCCCTCTCTCAAGTGC	GACACAGTCCTCGGCGTTCT
TaCOMT	ACGCTGCTCAAGAACTGCT	CGGGTTCACAGGCAGGAT
TaCAD	CTGCTCAAGGTGAACGGGAA	CATCATCTCCTGCGTCTCCTTC
